# Pathology of the Nutritive Changes in the Cartilages and of Acute Synovitis

**Published:** 1882-04

**Authors:** William Henry Porter

**Affiliations:** Professor of Surgical Pathology and General Surgery


### IX.—PATHOLOGY OF THE NUTRITIVE CHANGES IN THE CARTILAGES AND OF ACUTE SYNOVITIS: ONE OF A COURSE OF LECTURES DELIVERED AT THE COLUMBIA VETERINARY COLLEGE, N. Y., IN 1881–82.

BY WILLIAM HENRY PORTER, M.D., D.V.S.,
Professor of Surgical Pathology and General Surgery.

To-day, gentlemen, we commence the study of one of the most important departments of Surgical Pathology, viz.:

*Diseases of the Joints.*—First, let us consider some changes involving the cartilage alone, but not always producing a serious lesion of the joints, as a whole.

Like the bones, the cartilages are subject to structural changes. We have already learned that the cartilages contain no blood vessels, but receive their nourishment from the surrounding structures. Consequently, lesions of the cartilages may follow direct irritation or an arrest of the proper nutritive supply.

Changes brought about by irritation can easily be studied by experimenting upon permanent cartilage. Expose, for instance, a part of the surface of a cartilage, and at the end of two weeks we will find the exposed and injured surface covered over by a gray pulpy layer. If we make a thin section which will include both this pulpy layer and the cartilage beneath, the following conditions will be found in that portion of the cartilage which is furthest from the primary wound. The cartilage capsules contain cells, whose nuclei are easliy rendered visible, but as we approach the solution of continuity, the cells become larger than normal, and the protoplasm more abundant. The nutrition is increased, the cells become irritated, enlarged, and as a result the nucleus and its surrounding protoplasm tend to divide. In this way we soon have a distinct mass around each nucleus. This constitutes what is termed by some writers a formative irritation ; that is to say, the irritation has increased the number of cartilage cells. Each of these newly formed cells excretes around itself a cartilaginous substance, and thus forms a new and independent capsule. Up to this point, there have been only changes of structure and property which the cartilage cells nominally possess, but brought about more rapidly than naturally is the case. This zone of subdividing cartilage cells varies greatly in thickness.

As we approach the free surface, we find the cartilage substance broken into festoons, each excavation corresponding to an opened cartilage capsule, which has burst at its most superficial margin. By the side of these open capsules we find some that are unopened, which, however, are filled with young cells, which tend to destroy the formative property and ultimately cause their rupture.

The gray pulpy mass, visible to the naked eye, which we found upon the surface, consists of young and newly formed tissues, in which blood-vessels may be developed, taking their origin from the adjoining synovial membrane. This newly formed tissue, you will readily appreciate, has been developed from, and at the expense of the cartilage proper, which gradually disappears, and is replaced by this new material.

We have other changes in the cartilages which are secondary to a change in their nutritive supply, or the general nutrition of the system as a whole, namely: *Fatty degeneration*, *infiltration of urates*, *mucoid degeneration*, and *calcareous infiltration*.

I. Fatty degeneration should not be confounded with fatty infiltration, so common in old cartilages. Fatty degeneration causes death of the cell elements of the cartilage; so that, in place of the capsules and their contained cells, we find that they are replaced by fat granules. The intercellular substance is soft and often undergoes segmentation. These changes are primary lesions, and not secondary to a previous inflammation. In inflammation we have an opposite condition; that is, the few fat granules normally present, rapidly disappear.

II. We sometimes meet with a deposition of the crystals of urate of soda, magnesia, or lime; generally the former. The crystals in this affection are primarily deposited in the cartilage cells, and later may involve the fundamental substance between the cells. This gives rise to the condition known as gout, or gouty arthritis.

III. There is another condition of the cartilages which has been termed mucoid degeneration; which, however, is a normal physiological process in the costal cartilages, and is occasionally seen as an abnormal process in some of the joints. In this form of lesion the protoplasm of the cells undergoes a mucoid degeneration, which is generally accompanied by a segmentation of the basement substance.

IV. Occasionally we meet with infiltration of the cartilage with calcareous salt. This condition is the opposite of the infiltration of urate of soda, or gouty infiltration. In this lesion the inorganic salts are deposited first in the cartilage capsules, gradually extending into the intercellular substance; but the deposit never attacks the cartilage cells proper, in this respect having a distinct difference from the gouty lesion.

This histological point of deposition is of considerable practical and theoretical importance in comparative pathology, especially in sustaining the theories of gout and rheumatism. It has been held that gout was due to the incomplete assimilation of the nitrogenous foods, the rheumatism to the incomplete assimilation of the starches and fats—the former surcharging the blood with uric acid, which is followed by the deposit of the urates in various parts of the body. In the latter, it is supposed that the blood is surcharged with lactic acid, and, as a consequence, inflammation of the fibrous elements follows. Some seem inclined to think that both gout and rheumatism are the same, from the fact that in some of the lower animals and birds chalky deposits are found. I am inclined to think that these chalky deposits come under the head of calcareous infiltration, and not gouty infiltration, and until a number of microscopic examinations have been made to prove positively whether the capsule or the cell itself is primarily involved, it must remain an open question. All these changes are met with occasionally, and probably they are more frequent than is commonly supposed, owing to the lack of close observation.

Having considered these few trivial affections of the cartilage, independently of the joint as a whole, we are ready to study the lesions which may involve all the structures of the joint. Before commencing the study of these lesions (or articular diseases), we must first review briefly the structure of the most important parts which are included in the term "joint," and which go to form a normal articulation.

The cavities of all diarthrodial articulations are limited by the surface of cartilage covering the articulating extremity of the bones and the synovial membrane which is stretched from bone to bone, thus forming a shut sac. If a section is made through one of these joints they will always be found the same. If we take a section of of the cartilage and examine it from its free surface down to the bony connection, we find the following:

I. Along the free surface the cartilage capsules are lenticular or flattened.

II. A second layer made up of round capsules which, as a rule, contain only one cell, will be met with.

III. A third layer, which lies below the second, is composed of capsules which are lengthened perpendicularly to the surface, and each primary capsule contains two, three or more secondary capsules, lying directly behind each other.

These enlarged primary capsules form linear series which are continued into the deepest layer, or bottom, of the cartilage, where we find a narrow band, an infiltration of calcareous salts, which unite or cement, as it were, the cartilage to the underlying osseous tissue.

All the cartilage cells contained in the capsules of the first and middle layers normally contain granules, and even one or more fat droplets.

The calcified layer is bounded on the cartilage side by a sinuous line, on the bony side there are marked depressions and prominences, in the hollows of which fit papillary projections from the underlying bone, and *vice versa,* thus dovetailing the cartilage and bone together. In the centre of each of these osseous papillæ there is a medullary and vascular cavity, which communicate with the medullary and vascular spaces of the spongy or cancellated bone from which they spring. If the blood plasma reaches the cartilages from this side, it must of necessity pass directly through this osseous papillary and adjacent calcareous layer. These two layers being devoid of blood canals, it is argued by some writers that the cartilage receives no nutriment whatever from this side, but gains its whole nutrition solely from the fluid of the joint cavity. It seems quite probable, however, as is held by many, that the cartilage derives nourishment in part from all sides.

In studying the synovial membrane, we have a plain and a villous layer to consider. The plain layer is composed of a layer of fibrilated connective tissue interspersed with elastic fibres, and is in direct continuity with the perivascular connective tissue. This portion of the membrane is lined by a single layer of flattened connective-tissue corpuscles, called endothelial corpuscles, which closely resemble those seen on larger serous surfaces—as the pleura and peritoneum.

The villous surfaces, commonly called the synovial fringes, are best seen at points where the membrane is arranged in folds, in its passage from one surface to the opposite. The bases of these fringes are formed by the partial or complete opposition of the supporting layers of the synovial membrane proper. Between the two layers formed by the folding inward of the membrane we find a layer composed of loose connective tissue, adipose masses, and numerous blood-vessels which supply the fringes. As these fringes are very thin, they can be quite easily examined microscopically by separating them at the base and spreading the remaining portion on a slide, in glycerine and water, and covering it with a circle. If the fringe should chance to be congested, we will find the artery and vein distended and a very dense capillary network at their free border. From the extremity of these fringes we see bodies of various shapes projecting into the synovial cavity. Some are fusiform projections composed of an axis-cylinder of fibrillated connective tissue substance, covered by two or more layers of flattened cells. Prolongations frequently join them together which contain nuclei, so that they present a double contour. The cell bodies which cover these projections are almost identical in their appearance with those seen upon the choroid plexuses of the ventricles of the brain. Some of these papillary projections are club-shaped, their free extremity being much larger than the connecting stem. When they are club-shaped, the connecting central axis is much smaller than that of the straight variety. This form occasionally contains cartilage corpuscles. These cartilage corpuscles are a nucleus from which cartilaginous tumors spring, which may become detached and form a free foreign body in the joint cavity.

Vessels, nerves and lymphatics have been traced into the synovial membranes.

The synovial membrane does not cover the whole articular surface, but only a portion of the outer border. To just what point of the articular surface the membrane reaches is hard to determine, because it rapidly becomes thinner from the circumference to the centre, and blends with the perichondrium, soon becoming so thin that it cannot be positively traced.

The whole surface of the synovial membrane is not found completely covered by the so-called endothelial corpuscles. Only patches here and there are smooth and glistening in appearance, the intervening spaces being opaque.

The synovial fringes play a very important physiological function. The superficial cells have been supposed to secrete the synovial fluid, but it must come indirectly from the underlying blood-vessels.

The synovial fluid is rather complex in composition, as may be seen by this table, which gives the analysis of a number of observers :

| | I. | II. | III. | IV. | V. | VI. | VII.* |
|---|---|---|---|---|---|---|---|
| 1. Water. . . . . . | 98.03 | 94.00 | 96.57 | 96.99 | 94.85 | 92.83 | 92.80 |
| 2. Albuminous Matter. . . . | | | } 1.90 | } 1.57 | } 3.51 | 5.01 | 6.40 |
| 3. Albumen. . . . . | .53 | 3.50 | | | | | |
| 4. Extractive Matter. . . . . . . | | | | | | .38 | |
| 5. Gelatine. . . . . | .93 | | | | | | |
| 6. Mucin. . . . . . | | .50 | .32 | .24 | .56 | .65 | |
| 7. Fats. . . . . . . | | | .60 | .06 | .07 | .06 | |
| 8. Soluble Salts. . | | | } 1.06 | } 1.13 | | .87 | } .75 |
| 9. Insoluble Salts. | | | | | .99 | .04 | |
| 10. Muriate of Soda. | .23 | | | | | | |
| 11. Ash. . . . . . . | | 1.+ | | | | | |
| 12. Fixed Alkalies | Trace. | | | | | | |
| 13. Lime Phosphate | Trace. | | | | | | |
| | 99.72 | 99.+ | 99.91 | 99.99 | 99.98 | 99.84 | 99.95 |

From these different analyses, it is quite apparent that this fluid varies but little in its general composition.

If we examine a drop of this synovial fluid under the microscope, we find that it contains, in addition to fat molecules, a few amœboid corpuscles as well as cells similar to those which occur on the surface of the membrane. The phosphates of lime and soda are the principal salts.

Now that we are familiar with the structures entering into the formation of a normal articulation, we are ready to consider the pathological changes in the same.

*Joint Diseases* constitute the most important branch of surgical pathology for the veterinary surgeon to familiarize himself with.

Before studying diseases of the joint as a whole, let us consider, first, the pathology of inflammations which attack only the synovial membrane.

When we use the term synovitis, it can mean but one thing—an inflammation of the synovial membrane alone.

---

I *Davy, John.*

II. *Thudichum.* A Manual of Chemical Physiology. New York: 1872.

III. IV. V. } *Frerichs.* R. Wagner, Handwörterb, d. Physiologie, Bd 3, Abth, I. S.463.

VI. *Hoppe-Seyler.* Physiologische Chemie. Theil III, S. 623.

*VII *John.* Berzelius Lehrbuch übers. von Wöhler. 1831, Bd. IV. S, 461.

The synovial, like serous membranes, have the power of secreting a fluid called synovia, which is for the lubrication of the joints.

When an inflammation attacks this membrane, the quantity of synovia is greatly increased.

At the commencement of the inflammatory process, the vessels grow large and tortuous, the membrane loses its former lustre, and occasionally from the first it has a cloudy, yellow-reddish color; later, it becomes red and velvety. Upon the surface of the membrane we often find a layer of fibro-plastic matter, variable in thickness, similar to the plastic layer so commonly met with in pleuritic inflammations (by some called pseudo-membrane).

*Microscopically* the entire membrane or tissue is found infiltrated with plastic material; and upon the surface there is an abundant infiltration or collection of cells, giving the surface the appearance of a mass of granulations, the most superficial of which resemble closely pus cells. In the immediate vicinity of the distended blood-vessels, we find large quantities of wandering cells, which are undoubtedly the white-blood corpuscles which have escaped from the vessels and collected in the surrounding tissues. One peculiarity in synovitis is this, that the red-blood corpuscles, as well as the white, escape in great numbers, which is said to be an exception to the general rule of inflamed serous membranes.

The pseudo-membrane is composed entirely of small round cells, held together by coagulated fibrin. The normal striated or fibrillated appearance of the underlying connective tissue is less distinct, and now has a gelatinous or mucous consistency, and closely resembles granulation tissue.

The fluid of the joint is constantly becoming more and more cloudy, at first due to a few pus corpuscles, but later in the disease it becomes decidedly purulent.

In a short time the synovial membrane becomes so intensely congested that the distended capillaries are easily recognized by the naked eye, and the membrane somewhat resembles a sponge.

The membrane soon becomes nodulated and granular, representing a true granulating surface, from which pus is constantly being discharged. At this stage the inflammation may subside and the membrane be restored to its normal condition, but when prolonged beyond this point and suppuration continues, there will be a marked loss of substance, and the only possible method of recovery is in the formation of cicatricial tissue, which incompletely replaces that destroyed.

Histologically speaking, the result is a permanently impaired joint, but not necessarily a lame one. With a continuation of the synovitis, the cartilages become cloudy, and the various structures of the joint secondarily involved. Up to this point, however, we have been dealing with a lesion which is truly a synovitis; beyond this we must use the term arthritis, as the whole joint is now implicated. A pure and simple synovitis is a rare disease, but as a primary change in arthritis it is quite common, and the first which is readily recognized.

Before taking up injuries involving all the structures of the joint, we will consider those wounds which primarily involve the synovial membrane only. The synovial sac may be opened by external injury, while the other structures of the joint are not invaded. The joint is punctured in some way, either in the form of an incised or punctured wound. These forms of injuries are probably more frequent in the lower animals than in human beings.

Immediately following the receipt of the injury there is but little hemorrhage, but from the wound a fluid like the white of an egg escapes. In a case like this you may be certain that a joint cavity has been opened, otherwise the synovia could not have escaped. In the smaller joints the escape of synovia is so small that it is often unnoticed. Where we meet such cases it requires considerable skill to determine whether or not the joint has been opened. When fully satisfied that the wound has punctured the synovial membrane, the following rules should at once and invariably be observed: the animal, and especially the limb, should be kept as quiet as possible, and the joint absolutely at rest. The day will probably come when plaster-of-Paris, or, better still, tripolith, can be effectually applied to the injured limbs of the lower animals as in man.

Tripolith is a comparatively new substance for surgical dressings, receiving its name on account of its hardness and great power of resistance. It was discovered or brought before the public by Mr. B. Von Schenke, of Heidelberg.

The advantages claimed for tripolith are: First, it absorbs moisture from the air less freely than plaster-of-Paris. Second, tripolith is lighter. An equal volume of plaster and tripolith were weighed; the plaster weighed 604 grammes, tripolith 568 grammes. When dried the plaster weighed 470 grammes and the tripolith only 413 grammes. Thus, it will be seen that tripolith is about 14 per cent. lighter. Third, Tripolith hardens more quickly, plaster taking from fifteen minutes to one hour; the tripolith becomes perfectly set in five minutes. Fourth, tripolith when once hard remains unchanged by water, which enables a person, by preventing the entrance of water underneath the tripolith, to take the usual bath. Fifth, it is a trifle cheaper than plaster. If all the advantages claimed for this new material prove true, I see no reason why permanent and serviceable bandages cannot be applied to the lower animals as well as to man. In fact, I have heard of one case in which the hock joint was rendered immovable by plaster-of-Paris. The external wound should always be closed as quickly as possible, to prevent the escape of synovia, which has a great tendency to retard the healing of the wound and take away all chance of a primary union. There are a number of ways for doing this, the method to be governed by the size of the wound. The two principal ways, however, are to carefully shave off the hair immediately adjacent to the lips of the wound, and if the opening is small, cover it with flexible collodion; if large, a few interrupted sutures may be required; or, we can use both sutures and collodion. The essential thing, however, is to keep the joint perfectly quiet —immovable; and this can only be done by a well-applied plaster-of-Paris or tripolith bandage. If the patient has been highly fed prior to the receipt of the injury, we may find it advantageous to administer a mild purgative.

Some strongly advise the application of a few leeches, and at times you may be justified in so doing. Cold to parts, in the form of ice-bags, is sometimes of service. Some advocate warm applications.

In these wounds of the joints, I am convinced that by a well-applied bandage, rendering the joint perfectly immovable, far better results will be obtained than by any and all forms of antiphlogistic treatment. And the result will be many more speedy cures, and a much smaller number of permanently lame horses. In human surgery, the plaster-of-Paris method of treatment has wrought such wonderful results as to gain the title of very brilliant surgery, and it certainly will be doubly brilliant when made a success in the daily practice of every veterinarian.

About the third or fourth day after the receipt of the injury there is burning pain in the joint, slight fever, and we find the affected joint warmer than the opposite. On the fifth or sixth day remove your sutures, for they have done all the good they can and are now a source of irritation.

From this point the case may terminate in one of two ways.

First, and most favorably (which, however, is the most frequent, when treated in an immovable apparatus), is in a speedy recovery. The wound heals, perhaps, by first intention; the swelling, pain and heat have all subsided, so that at the end of three or four weeks, when the dressings are removed, you will find the joint movable, and a speedy recovery usually follows.

In the more unfavorable cases, the supposition is that the case does not come under treatment until some days, or it may be weeks, after the receipt of the injury. In such cases we find great swelling and heat in and around the joint, and the whole limb is often œdematous; there is severe pain when the limb is handled or moved; toward evening the temperature rises; the appetite fails, and the patient rapidly becomes emaciated.

Now, if you pay no attention to the limb, it will gradually become flexed, and if recovery follows, the patient may be totally unable to place the foot on the ground. There are various theories as to why the joint becomes flexed; First, that the irritation to the sympathetic or sensory nerves carries the stimuli to the origin of motor activity, and the flexors thus become most inflamed; second, that a joint will contain more fluid when semi-flexed. Another explanation, and one which seems most rational, is the simple fact that in a state of natural rest the joints are prone to fall into a flexed or semi-flexed condition, and again, when muscular activity is interfered with, it is most likely to be primary on the extensor side and not on the flexor. The flexor, therefore, being unimpaired, would tend to produce the semi-flexion.

When these symptoms of inflammation in the aggravated cases have developed, antiphlogistic remedies assume their historic value; but, above all things, the position of the limb must not be forgotten, for in case the joint should become stiff, or anchylosed, you want the limb in a position where it will be serviceable, which at best will be poor enough in the lower animals. You will remember, I

think, that we found that the tendency was for the limb to become semi-flexed, but in your practice, as in the lower extremity of man, if the joint is to become stiff, or anchylosed, the most favorable position would be that of extension or nearly straight. In the elbow joint of man, the flexed position is the one to be desired.

The best method of treatment for these severely inflamed joints is rest, cold applications and the local use of Tr. Iodin. Comp.

It sometimes happens that the fluid in the joint increases in quantity very rapidly, giving rise to insupportable tension when there is no free escape by the original wound, which fluid is quite purulent. At this point there is great danger from ulceration and breaking down of the capsule of the joint from within, and a flowing of the fluid into the surrounding cellular tissue, and a diffuse cellular inflammation.

To avoid this accident, we must puncture the joint either with a trochar or aspirating needle and draw off the fluid, always taking great care that no air enters the joint. Tapping of joint cavities has been repeatedly practiced in human surgery, and the disease found to terminate in a complete recovery, even at this stage. Since the use of the plaster-of-Paris splints, this extensive swelling, necessitating the tapping of the joint has been overcome in almost every case. There may be a complete recovery even after the synovial membrane has been ulcerated through, but generally there is more or less permanent stiffness of the joint.

This form of disease, however, frequently passes into a more chronic condition, the suppurating process attacking the tissues more deeply, but if recovery follows, there will always be more or less stiffness.

